# ZC3HC1 has functions distinct from TPR and is dispensable for TPR localisation to the nuclear basket

**DOI:** 10.12688/wellcomeopenres.23711.1

**Published:** 2025-04-11

**Authors:** Bethany M Bartlett, Juan Carlos Acosta, Wendy A Bickmore

**Affiliations:** 1 MRC Human Genetics Unit, Institute of Genetics and Cancer, University of Edinburgh, Edinburgh, Scotland, EH42XU, UK; 2 Institute of Biomedicine and Biotechnology of Cantabria, CSIC-Universidad de Cantabria, Santander, 39011, Spain

**Keywords:** histone mRNA export, mRNA export. nuclear pore

## Abstract

**Background:**

The nuclear basket is a ‘fishtrap’-like structure on the nucleoplasmic face of the nuclear pore complex which has been implicated in diverse functions including RNA export, heterochromatin organisation, and mitosis. Recently, a novel component of the nuclear basket, ZC3HC1, has been described. The localisation of ZC3HC1 to nuclear pores has been reported to occur reciprocally with TPR, a major structural component of the nuclear basket.

**Methods:**

Using siRNA-mediated knock down, immunofluorescence and RNA sequencing we compare the consequences of depleting two proteins of the nuclear pore basket – TPR and ZC3HC1.

**Results:**

We show that in human fibroblasts, although ZC3HC1 localisation to nuclear pores is TPR-dependent, TPR localises to pores regardless of the presence of ZC3HC1. We demonstrate that knockdown of TPR and ZC3HC1 produce distinct transcriptional profiles.

**Conclusions:**

Our results suggest that there is little overlap in function between these two nuclear basket proteins in human diploid fibroblasts.

## Plain language summary

Nuclear pores are structures critical for the regulating how molecules get into and out of the nucleus of our cells. They provide a channel through which selected cargoes are transported. These cargoes include the messenger RNAs that carry the information from DNA in the nucleus to make proteins in the cell cytoplasm. On the nuclear side of the nuclear pore, there is a poorly understood structure called the nuclear basket. This paper explores two proteins called TPR and ZC3HC1 that localise at the nuclear basket. Exploring the function of these two proteins, this paper shows that whilst TPR is important for the export of certain classes of messenger RNA, ZC3HC1 is not. This suggests that these two proteins of the nuclear basket have quite different functions. In addition, we show that, whilst ZC3HC1 depends on TPR for its localisation at nuclear pores, the reverse is not the case - TPR localisation appears to be independent of ZC3HC1.

## Introduction

The nuclear pore complex (NPC) is a large transmembrane complex consisting of around 30 different proteins known as nucleoporins (Nups), organised into a cylindrical assembly with eightfold symmetry ( Petrovic *et al.*, 2022). The core structure consists of the inner ring, which lines the lumen of the nuclear pore, and outer rings sitting on each side of the nuclear envelope. On the cytoplasmic face of the pore, eight filaments extend into the cytoplasm, and on the nuclear side, eight nucleoplasmic filaments are joined to a double nuclear ring, forming a structure known as the nuclear basket ( Singh *et al.*, 2024).

**Figure 1.**
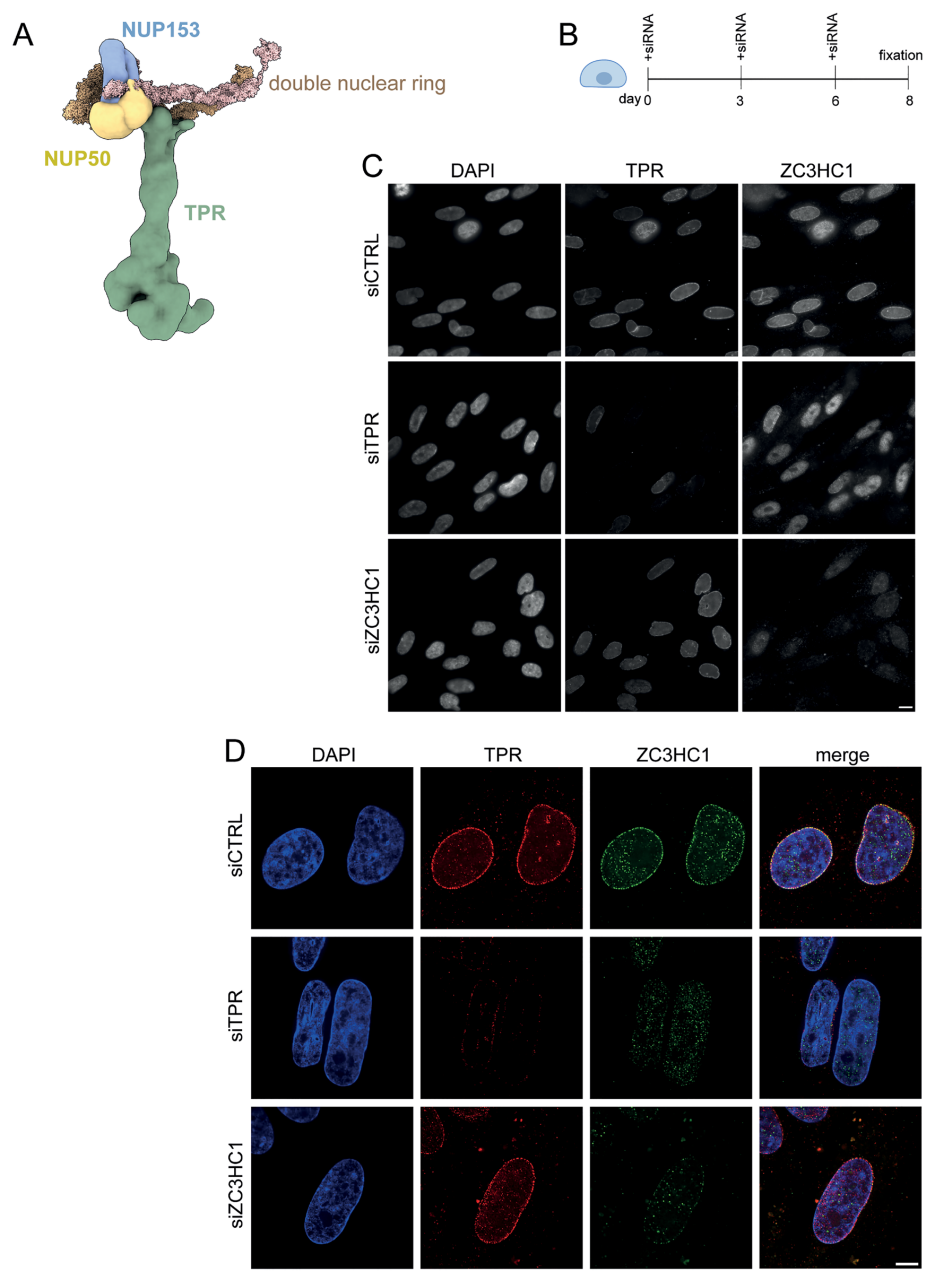
ZC3HC1 localisation to nuclear pores is dependent on TPR but ZC3HC1 knockdown does not delocalise TPR. **A**) Model of the mammalian nuclear pore basket showing the position of TPR (green), NUP50 (yellow), NUP153 (blue) and the double nuclear ring (brown). Adapted, with permission from Singh *et al.*, 2024. **B**) Schematic of experimental protocol for ZC3HC1 knockdown in IMR90 cells. **C**) TPR and ZC3HC1 immunostaining in DAPI-stained IMR90 cells after treatment for 8 days with control (CTRL) siRNA or with siRNAs targeting TPR or ZC3HC1. Immunostaining was carried out with antibodies detecting TPR (Abcam ab84516) or ZC3HC1 (Santa Cruz sc-365058). **D**) As in ( **C**) but imaged on a SoRa spinning disk confocal microscope. Immunostaining was carried out with antibodies detecting TPR or ZC3HC1 that were a gift from Volker Cordes and Philip Gunkel ( Table 1). Scale bars: 10μm.

The nuclear basket was first described as a ‘fishtrap’-like structure attached to the NPC ( Maul, 1976). Although there are near-atomic structures of the rest of the NPC, until recently the position of proteins in the nuclear basket had only been coarsely approximated ( Allegretti *et al.*, 2020; Kim *et al.*, 2018; Niepel *et al.*, 2013). However, cryo-electron tomography (cryo-ET) and integrative structural modelling have now provided unprecedented understanding of how the nuclear basket docks on to the double nuclear rings of the mammalian NPC ( Singh *et al.*, 2024) ( Figure 1A).

The nuclear basket has a range of functions, many of which have been attributed to TPR, a 267-kDa basket Nup which is anchored to the NPC by its interaction with NUP153 ( Hase & Cordes, 2003). TPR is required for the specific export of short or intronless mRNAs by the TREX-2 complex ( Aksenova *et al.*, 2020; Lee *et al.*, 2020; Zuckerman *et al.*, 2020). TPR also has a role in heterochromatin organisation – it is necessary for the exclusion of heterochromatin at nuclear pores, and for the formation of senescence-associated heterochromatic foci (SAHF) in senescent cells ( Boumendil *et al.*, 2019; Krull *et al.*, 2010). During oncogene-induced senescence, TPR is also necessary for inflammatory signalling, which we recently showed is due to the role of TPR in the generation of cytoplasmic chromatin fragments ( Bartlett *et al.*, 2024; Boumendil *et al.*, 2019).

Until recently the metazoan nuclear basket was thought to consist only of TPR and the NUP153 and NUP50 anchors to the nuclear ring ( Krull *et al.*, 2004; Lin & Hoelz, 2019) and the relative position of these proteins is confirmed by the recent molecular structure study ( Singh *et al.*, 2024) ( Figure 1A). More recently, another component, ZC3HC1, was found in isolated *Xenopus* oocyte nuclear envelopes, and subsequently confirmed to be part of the nuclear basket in other cells, including human cell lines, by electron microscopy ( Gunkel *et al.*, 2021). ZC3HC1, also known as NIPA, was previously shown to be part of a SCF E3 ubiquitin ligase which promotes the degradation of cyclin B1 during the cell cycle ( Bassermann *et al.*, 2005). TPR and ZC3HC1 are reported to show reciprocally dependent localisation to the NPC ( Gunkel *et al.*, 2021), with two pools of TPR in the nucleus; one which depends on ZC3HC1 for localisation to the nuclear pore and one which is ZC3HC1 independent ( Gunkel & Cordes, 2022). ZC3HC1 interacts with the NPC via its nuclear basket interaction domain, which is made up of two zinc-finger containing modules ( Gunkel *et al.*, 2023). A yeast homolog of ZC3HC1, known as Pml39, has a nuclear basket interaction domain with a similar structure to the human protein, but a low degree of sequence similarity ( Gunkel *et al.*, 2023). Pml39 is involved in the retention of improper messenger ribonucleoparticles in the nucleus ( Palancade *et al.*, 2005), suggesting that ZC3HC1 could play a role similar to TPR in regulating mRNA export. The precise position of Pml39/ZC3HC1 in the nuclear basket has yet to be determined ( Singh *et al.*, 2024)

Here we show that, in primary human fibroblasts, although ZC3HC1 localises at the NPC and that this localisation depends on TPR, the localisation of TPR to nuclear pores is not ZC3HC1 dependent, contrary to previous reports. Furthermore, knockdown of ZC3HC1 produces a very different transcriptional signature to TPR knockdown, suggesting that the two proteins have different functions.

## Methods

### Cell culture and siRNA transfection

Human IMR90 cells, infected with pLNC-ER:STOP retroviral vectors to produce neomycin resistant control cells ( Acosta *et al.*, 2013), were cultured in DMEM with 10% FBS, 100 nM 4-hydroxytamoxifen and 1% penicillin/streptomycin in a 37 ^o^C incubator with 5% CO _2_.

siRNA knockdown was carried out as previously described ( Bartlett *et al.*, 2024; Boumendil *et al.*, 2019). Briefly, 9 × 10 ^5^ STOP IMR90 cells (except for imaging experiments, which used 1.5 × 10 ^5^ cells) were transfected using Dharmafect transfection reagent (Dharmacon) with a 30nM final concentration of control (siCTRL, D-001810-10-59) or TPR (siTPR, L-010548-00) or ZC3HC1 (siZC3HC1, L-016879-02) siRNA pools (Dharmacon). Transfections were carried out in the presence of 4-OHT and were repeated at day 0, 3 and 6 and cells fixed for imaging on day 8 ( Figure 1B). For RNA-seq, cells were harvested after 3 days after siRNA transfection.

### Immunofluorescence

Cells grown on coverslips were fixed with 4% paraformaldehyde and blocked with 1% bovine serum albumin (BSA) as previously described ( Bartlett *et al.*, 2024). Coverslips were then incubated with primary antibody diluted in 1% BSA at the dilutions detailed in Table 1, for 45 mins in a humid chamber. After washing three times with PBS, coverslips were then incubated for 30 mins with fluorescently labelled secondary antibodies (Life Technologies, Table 1) followed by two washes in PBS. PBS with 50ng/ml DAPI was added for 4 mins, before a final wash with PBS and mounting onto slides with VectaShield (Vector Laboratories).

**Table 1.** List of antibodies. Antibodies used in immunofluorescence (IF) experiments with their corresponding dilutions. RRIDs are from https://www.rrids.org

	Source or reference	Identifiers	Dilution
anti-TPR (rabbit polyclonal)	Abcam. Raised against residues 2300–2349	ab84516 RRID:AB_1861454	IF (1:500)
anti-TPR (mouse monoclonal)	Gift from Volker Cordes. Raised against residues 1462–1500		IF (1:100)
anti-ZC3HC1 (mouse monoclonal)	Santa Cruz. Raised against residues 1–300	sc-365058 RRID:AB_10847677	IF (1:100)
anti-ZC3HC1 (guinea pig polyclonal)	Gift from Volker Cordes. Raised against residues 307–355		IF (1:200)
anti-mouse IgG (H+L) secondary, Alexa Fluor 568 (donkey polyclonal)	Invitrogen	A10037 RRID:AB_11180865	IF (1:1000)
anti-rabbit IgG (H+L) secondary, Alexa Fluor 488 (goat polyclonal)	Invitrogen	A11034 RRID:AB_2576217	IF (1:1000)

Epifluorescence images were acquired as previously described ( Bartlett *et al.*, 2024). Super-resolution images were acquired by Instant Sim microscopy ( Azuma & Kei, 2015) using a Nikon SoRa™ system. Imaging was carried out using a SR HP Plan Apo λS 100x 1.35NA Silicone lens (Nikon Instruments). The CMOS cameras used for acquisition were Teledyne Photometrics Prime 95B 488 / 561nm laser lines. Step size for Z stacks was set to 0.120μm as required by manufacturer’s software. Acquisition of images and deconvolution was carried out using the Nikon NIS Elements Advanced Research software ( https://www.microscope.healthcare.nikon.com/products/software/nis-elements/software-resources).

### RNA-seq library preparation and analysis

Total RNA was extracted from a 10cm tissue culture plate using the RNeasy mini kit (Qiagen). Library preparation, sequencing and data quality control were carried out as previously described ( Bartlett *et al.*, 2024).

Differential expression analysis was carried out using DeSeq2 ( Love *et al.*, 2014). Gene ontology analysis was carried out using clusterProfiler ( Wu *et al.*, 2021). Volcano plots were rendered using ggplot2 ( Wickham, 2016) and Venn diagrams rendered using VennDiagram ( Chen & Boutros, 2011). A list of intronless genes was obtained from the UCSC hg19 GTF file ( Nassar *et al.*, 2023) by sorting for genes with a single exon. The list of histone genes was obtained from HGNC ( Braschi *et al.*, 2019).

### Statistics

Statistical analysis was performed using R and the specific statistical tests used are described in the relevant text and Figure legends. p-value significance is denoted as follows: * < 0.05, **< 0.01, *** < 0.001.

### Data availability

RNA-seq data for TPR and ZC3HC1 are available from NCBI GEO under Accession numbers GSE264387 and GSE286436 respectively.

## Results

### ZC3HC1 localisation to nuclear pores is dependent on TPR but ZC3HC1 knockdown does not delocalise TPR

We first sought to verify the presence of ZC3HC1 at nuclear pores. We used siRNAs to deplete TPR or ZC3HC1 in human IMR90 fibroblasts ( Bartlett *et al.*, 2024) over an 8-day period ( Figure 1B). Immunofluorescence and wide-field epifluorescence microscopy showed both TPR and ZC3HC1 present at the nuclear periphery in cells transfected with control siRNA ( Figure 1C). Depleting TPR caused ZC3HC1 to move away from the nuclear periphery and into the nucleoplasm as previously reported ( Gunkel *et al.*, 2021). However, TPR remained localised at the nuclear periphery in cells depleted of ZC3HC1. Super-resolution microscopy showed TPR and ZC3HC1 colocalised at nuclear pores in cells treated with the control siRNA ( Figure 1D). Although TPR knockdown caused ZC3HC1 to move away from the nuclear periphery, TPR remained visibly localised to nuclear pores when ZC3HC1 was knocked down ( Figure 1D).

### The transcriptional signatures of ZC3HC1 and TPR knockdown are distinct

To better differentiate the functions of ZC3HC1 and TPR, we examined whether the transcriptional changes upon ZC3HC1 knockdown are similar to those that we have reported upon knockdown of TPR in IMR90 cells ( Bartlett *et al.*, 2024). We carried out RNA-seq on IMR90 fibroblasts treated with either control (siCTRL) or ZC3HC1 siRNAs for three days, using the same protocol as for TPR knockdown ( Bartlett *et al.*, 2024).

**Figure 2.**
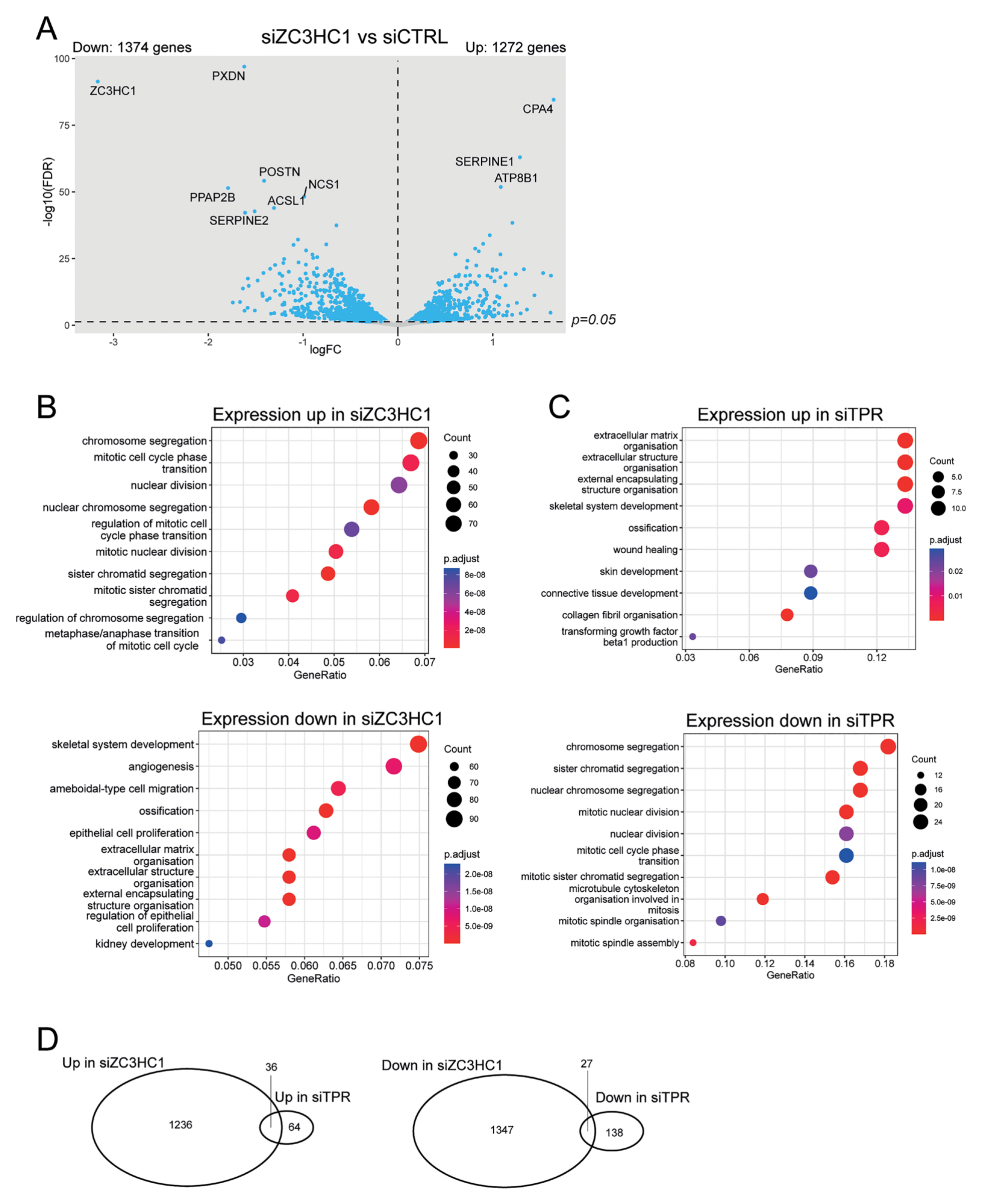
TPR and ZC3HC1 knockdown have distinct transcriptional signatures. **A**) Volcano plots showing differential expression analysis comparing siZC3HC1 and siCTRL samples. IMR90 cells were treated with the indicated siRNAs for 3 days. Blue dots indicate differentially expressed genes (adjusted p-value (FDR) < 0.05). The dashed horizontal line indicates an adjusted p-value of 0.05 and the dashed vertical line indicates a logFC of 0. The 10 genes with the most significant p-values are labelled. **B** and **C**) GO analysis carried out using clusterProfiler ( Wu *et al.*, 2021) for genes which increase or decrease in expression when ( **B**) ZC3HC1 or ( **C**) TPR is knocked down. RNA-seq data for TPR knockdown is taken from ( Bartlett *et al.*, 2024). **D**) Venn diagrams representing the overlaps between significantly up or downregulated transcripts upon TPR or ZC3HC1 loss in IMR90 cells.

ZC3HC1 knockdown led to more extensive changes in gene expression than knocking down TPR ( Bartlett *et al.*, 2024), with 1272 genes upregulated and 1374 downregulated upon ZC3HC1 knockdown ( Figure 2A). The most downregulated gene was *ZC3HC1*, confirming successful knockdown. Gene ontology analysis showed significant differences between genes whose expression changes in response to either ZC3HC1 or TPR knockdown ( Figure 2B and C). Loss of ZC3HC1 led to upregulation of genes associated with chromosome segregation and mitosis, consistent with the known role of ZC3HC1 in degrading cyclin B1 ( Bassermann *et al.*, 2005). Genes associated with angiogenesis, skeletal system development and extracellular matrix organisation were downregulated upon ZC3HC1 knockdown ( Figure 2B). TPR knockdown showed the opposite: mRNAs for genes involved in chromosome segregation were downregulated and those associated with extracellular matrix organisation were upregulated ( Figure 2C).

To investigate whether the two nuclear basket proteins have any shared functions, we examined how many genes change in expression upon both TPR knockdown and ZC3HC1 knockdown. Thirty-six (2.8%) of the genes upregulated upon ZC3HC1 knockdown were also upregulated on TPR knockdown, while 27 (2.0%) of the genes downregulated upon ZC3HC1 knockdown were also downregulated on TPR knockdown ( Figure 2D). The limited overlap in differentially expressed genes between the two knockdown experiments suggests that TPR and ZC3HC1 have distinct functions.

### ZC3HC1 is not required for the export of mRNAs from intronless genes

**Figure 3.**
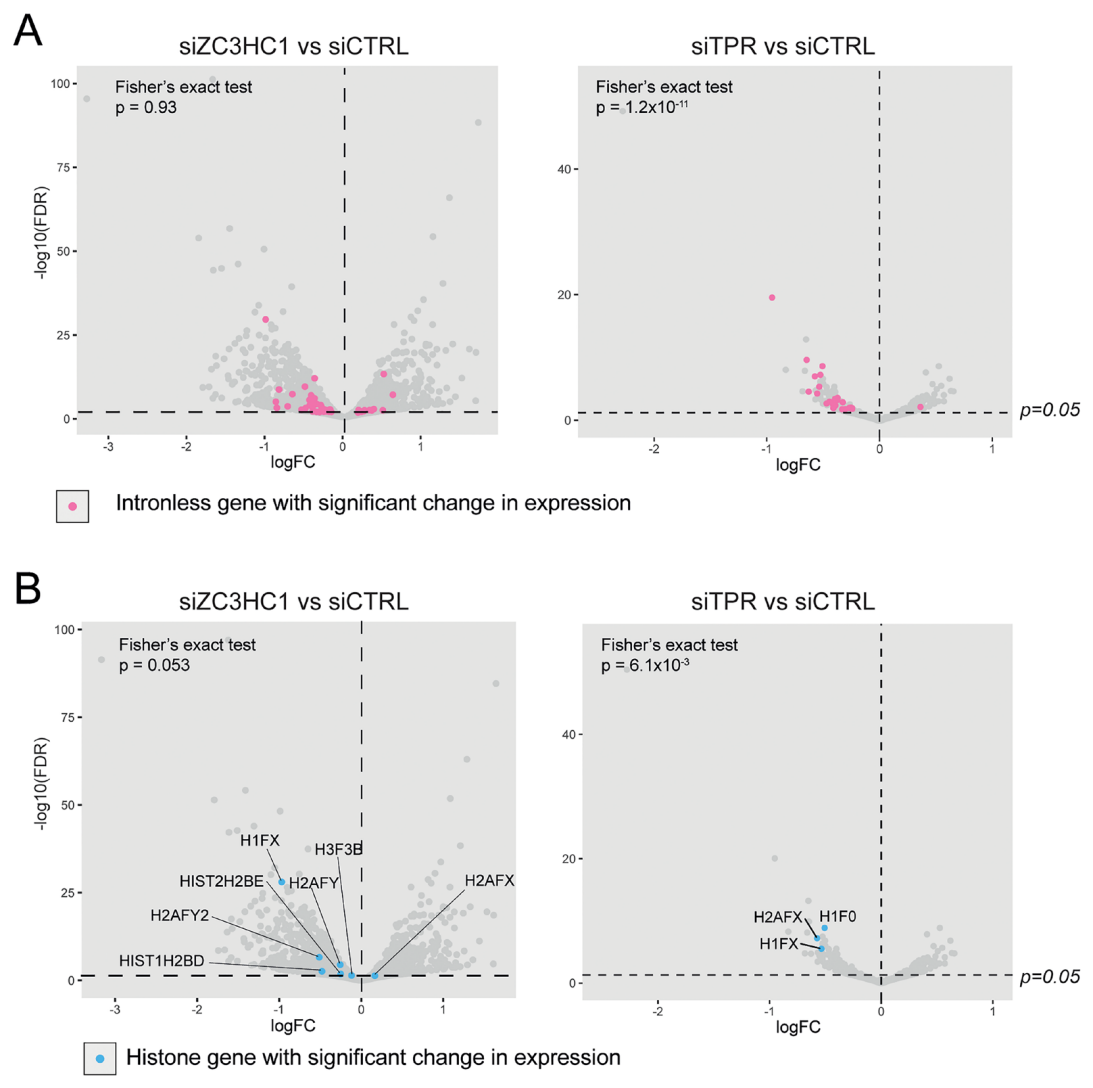
ZC3HC1 knockdown does not affect the expression of intronless or histone genes. **A**) Volcano plots of differential expression analysis of IMR90 cells treated with CTRL vs ZC3HC1 siRNAs (left), or CTRL vs TPR siRNAs (right). Intronless genes are labelled in pink. The horizontal dashed line indicates an adjusted p-value (FDR) of 0.05 and the vertical dashed line indicates a logFC of 0. Fisher’s exact tests were carried out to determine whether the number of downregulated intronless genes was greater than expected by chance. **B**) As in ( **A**) but with histone genes labelled in blue. Fisher’s exact tests were carried out to determine whether the number of downregulated histone genes was greater than expected by chance.

Consistent with the known role of TPR in the nuclear export of short and intronless mRNAs via interaction with the TREX-2 complex, TPR knockdown causes significant downregulation of mRNAs from intronless and histone genes ( Aksenova *et al.*, 2020; Bartlett *et al.*, 2024; Lee *et al.*, 2020; Zuckerman *et al.*, 2020). We investigated whether ZC3HC1, by localizing TPR at the nuclear basket, might play a similar role in mRNA nuclear export. Fisher’s exact tests showed that, in contrast to TPR knockdown, there were no more mRNAs for intronless genes significantly downregulated upon ZC3HC1 knockdown than would be expected by chance (p=0.93) ( Figure 3A). There were also no more histone mRNAs downregulated upon ZC3HC1 knockdown than would be expected by chance (p=0.053) ( Figure 3B). This suggests that, unlike TPR, ZC3HC1 does not have a role in the nuclear export of intronless mRNAs.

## Discussion

We have confirmed that ZC3HC1 localises to the nuclear periphery, suggesting that it is a nuclear pore protein, as reported ( Gunkel *et al.*, 2021). However, depleting ZC3HC1 in IMR90 cells did not affect the localisation of TPR to nuclear pores, as assessed by immunofluorescence, contrary to previous reports ( Gunkel *et al.*, 2021; Gunkel & Cordes, 2022). This suggests that, at least in human fibroblasts, ZC3HC1 may not play the structural role in establishing interconnections between TPR polypeptides at the nuclear basket which has been reported in cancer cells ( Gunkel & Cordes, 2022).

We did confirm that TPR is required for the localisation of ZC3HC1 to nuclear pores in IMR90 fibroblasts ( Figure 1). Even though ZC3HC1 is displaced to the nucleoplasm on TPR knockdown, only a small number of mRNAs which we previously reported changed in expression after TPR knockdown ( Bartlett *et al.*, 2024) also change in expression upon ZC3HC1 knockdown ( Figure 2). This suggests that most mRNAs that change in expression in response to reduction of ZC3HC1 do not depend on its localisation at the nuclear basket. Amongst the mRNAs downregulated upon ZC3HC1 depletion, we do not see an enrichment of mRNAs originating from intronless or histone genes, which are known to dependent on TPR localisation at the nuclear pore basket for their nuclear export ( Aksenova *et al.*, 2020; Bartlett *et al.*, 2024; Lee *et al.*, 2020) ( Figure 3). This further supports our conclusion that ZC3HC1 is not required for TPR localisation to the nuclear pore basket in IMR90 cells.

ZC3HC1 has been reported as a component of a nuclear SCF E3 ligase, which is required for the degradation of cyclin B1 and thereby regulates mitotic entry and exit ( Bassermann *et al.*, 2005). Our RNA-seq data showing that levels of mRNAs involved in chromosome segregation and the mitotic cell-cycle phase are elevated on ZC3HC1 knockdown is consistent with a role for ZC3HC1 in the regulation of mitosis. To distinguish which functions of ZC3HC1 are dependent on its localisation to the nuclear pore, RNA-seq could be repeated in cells expressing a version of ZC3HC1 which cannot localise to nuclear pores, for example by making a single amino acid substitution in ZC3HC1 (C429S) which abolishes its interaction with TPR ( Gunkel & Cordes, 2022).

## Ethics and consent

Ethical approval and consent were not required

## Data Availability

All quantitative data associated with this manuscript are been deposited in NCBI GEO and is freely accessible with no restrictions on use or distribution. NCBI GEO: RNA-seq data following TPR knockdown in IMR90 cells. GSE264387; https://www.ncbi.nlm.nih.gov/geo/query/acc.cgi?acc=GSE264387 ( Bartlett *et al.*, 2024) NCBI GEO: RNA-seq data following ZC3H1 knockdown in IMR90 cells. GSE286436; https://www.ncbi.nlm.nih.gov/geo/query/acc.cgi?acc=GSE286436 ( Bartlett & Bickmore, 2025)
