# ZC3HC1 has many functions distinct from TPR and is dispensable for TPR localisation to the nuclear basket

**DOI:** 10.12688/wellcomeopenres.23711.2

**Published:** 2025-11-24

**Authors:** Bethany M Bartlett, Juan Carlos Acosta, Wendy A Bickmore

**Affiliations:** 1 MRC Human Genetics Unit, Institute of Genetics and Cancer, University of Edinburgh, Edinburgh, Scotland, EH42XU, UK; 2 Institute of Biomedicine and Biotechnology of Cantabria, CSIC-Universidad de Cantabria, Santander, 39011, Spain

**Keywords:** histone mRNA export, mRNA export. nuclear pore

## Abstract

**Background:**

The nuclear basket is a ‘fishtrap’-like structure on the nucleoplasmic face of the nuclear pore complex which has been implicated in diverse functions including RNA export, heterochromatin organisation, and mitosis. Recently, a novel component of the nuclear basket, ZC3HC1, has been described. The localisation of ZC3HC1 to nuclear pores has been reported to occur reciprocally with TPR, a major structural component of the nuclear basket.

**Methods:**

Using siRNA-mediated knock down, immunofluorescence and RNA sequencing we compare the consequences of depleting two proteins of the nuclear pore basket – TPR and ZC3HC1.

**Results:**

We show that in human fibroblasts, although ZC3HC1 localisation to nuclear pores is TPR-dependent, TPR remains localised to pores when ZC3HC1 is depleted. Consistent with this, during oncogene-induced senescence, knockdown of ZC3HC1 does not compromise the formation of senescence-associated heterochromatin foci or activation of the senescence-associated secretory phenotype, which are both known to depend on the presence of TPR at the nuclear basket. We demonstrate that knockdown of TPR and ZC3HC1, although partially overlapping, also have many distinct transcriptional features

**Conclusions:**

Our results suggest that there is limited overlap in function between these two nuclear basket proteins in human diploid fibroblasts.

## Plain language summary

Nuclear pores are structures critical for the regulating how molecules get into and out of the nucleus of our cells. They provide a channel through which selected cargoes are transported. These cargoes include the messenger RNAs that carry the information from DNA in the nucleus to make proteins in the cell cytoplasm. On the nuclear side of the nuclear pore, there is a poorly understood structure called the nuclear basket. This paper explores two proteins called TPR and ZC3HC1 that localise at the nuclear basket. Exploring the function of these two proteins, this paper shows that whilst TPR is important for the export of certain classes of messenger RNA, ZC3HC1 is not. This suggests that these two proteins of the nuclear basket have quite different functions. In addition, we show that, whilst ZC3HC1 depends on TPR for its localisation at nuclear pores, the reverse is not the case - TPR localisation appears to be independent of ZC3HC1.

## Introduction

The nuclear pore complex (NPC) is a large transmembrane complex consisting of around 30 different proteins known as nucleoporins (Nups), organised into a cylindrical assembly with eightfold symmetry ( Petrovic *et al.*, 2022). The core structure consists of the inner ring, which lines the lumen of the nuclear pore, and outer rings sitting on each side of the nuclear envelope. On the cytoplasmic face of the pore, eight filaments extend into the cytoplasm, and on the nuclear side, eight nucleoplasmic filaments are joined to a double nuclear ring, forming a structure known as the nuclear basket ( Singh *et al.*, 2024).

**Figure 1.**
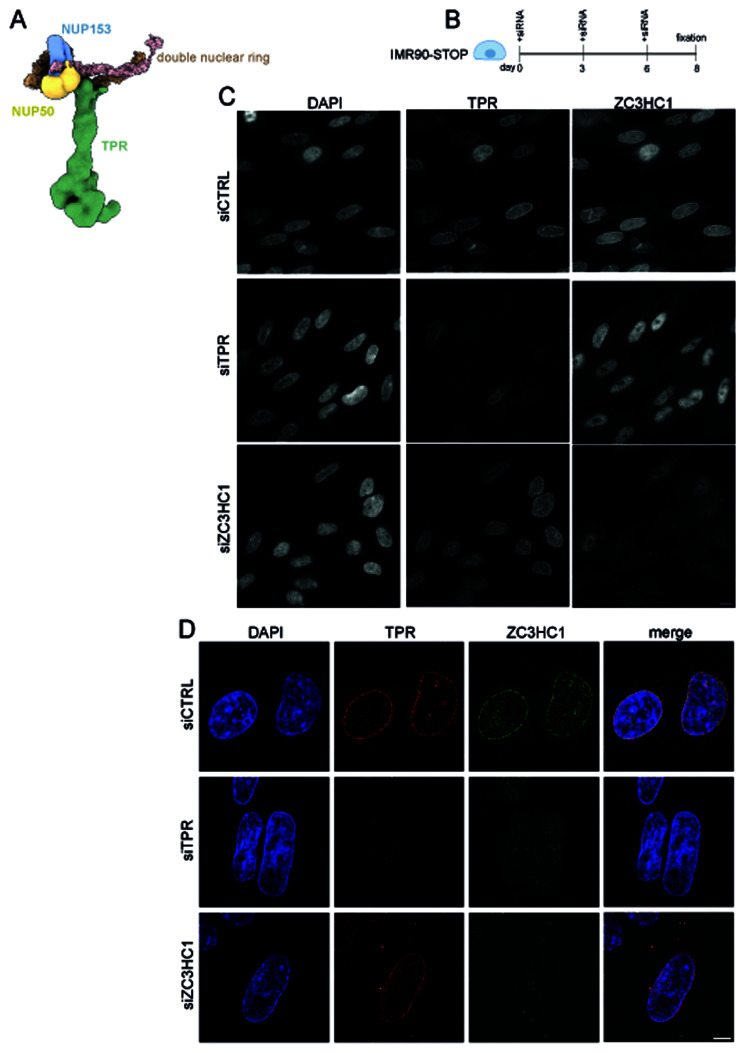
ZC3HC1 localisation to nuclear pores is dependent on TPR but ZC3HC1 knockdown does not delocalise TPR. **A**) Model of the mammalian nuclear pore basket showing the position of TPR (green), NUP50 (yellow), NUP153 (blue) and the double nuclear ring (brown). Adapted, with permission from Singh *et al.*, 2024. **B**) Schematic of experimental protocol for ZC3HC1 knockdown in IMR90-STOP cells. **C**) TPR and ZC3HC1 immunostaining in DAPI-stained IMR90-STOP cells after treatment for 8 days with control (CTRL) siRNA or with siRNAs targeting TPR or ZC3HC1. Immunostaining was carried out with antibodies detecting TPR (Abcam ab84516) or ZC3HC1 (Santa Cruz sc-365058). **D**) As in ( **C**) but imaged on a SoRa spinning disk confocal microscope. Immunostaining was carried out with antibodies detecting TPR or ZC3HC1 that were a gift from Volker Cordes and Philip Gunkel ( Table 1). Scale bars: 10μm.

The nuclear basket was first described as a ‘fishtrap’-like structure attached to the NPC ( Maul, 1976). Although there are near-atomic structures of the rest of the NPC, until recently the position of proteins in the nuclear basket had only been coarsely approximated ( Allegretti *et al.*, 2020; Kim *et al.*, 2018; Niepel *et al.*, 2013). However, cryo-electron tomography (cryo-ET) and integrative structural modelling have now provided unprecedented understanding of how the nuclear basket docks on to the double nuclear rings of the mammalian NPC ( Singh *et al.*, 2024) ( Figure 1A).

The nuclear basket has a range of functions, many of which have been attributed to TPR, a 267-kDa basket Nup which is anchored to the NPC by its interaction with NUP153 ( Hase & Cordes, 2003). TPR is required for the specific export of short or intronless mRNAs by the TREX-2 complex ( Aksenova *et al*., 2020; Lee *et al*., 2020; Zuckerman *et al*., 2020) and appears to enhance the frequency at which mRNPs enter the pore for export ( Li *et al*., 2021). TPR also has a role in heterochromatin organisation – it is necessary for the exclusion of heterochromatin at nuclear pores, and for the formation of senescence-associated heterochromatic foci (SAHF) in senescent cells ( Boumendil *et al.*, 2019; Krull *et al.*, 2010). During oncogene-induced senescence, TPR is also necessary for inflammatory signalling, which we recently showed is due to the role of TPR in the generation of cytoplasmic chromatin fragments ( Bartlett *et al.*, 2024; Boumendil *et al.*, 2019).

Until recently the metazoan nuclear basket was thought to consist only of TPR and the NUP153 and NUP50 anchors to the nuclear ring ( Krull *et al.*, 2004; Lin & Hoelz, 2019) and the relative position of these proteins is confirmed by the recent molecular structure study ( Singh *et al*., 2024) ( Figure 1A). More recently, another component, ZC3HC1 (previously also known as NIPA), was found in isolated *Xenopus* oocyte nuclear envelopes, and subsequently confirmed to be part of the nuclear basket in other cells, including human cell lines, by electron microscopy ( Gunkel *et al.*, 2021). ZC3HC1, also known as NIPA, was previously shown to be part of a SCF E3 ubiquitin ligase which promotes the degradation of cyclin B1 during the cell cycle ( Bassermann *et al.*, 2005). TPR and ZC3HC1 are reported to show reciprocally dependent localisation to the NPC ( Gunkel *et al.*, 2021), with two pools of TPR in the nucleus; one which depends on ZC3HC1 for localisation to the nuclear pore and one which is ZC3HC1 independent ( Gunkel & Cordes, 2022). ZC3HC1 interacts with the NPC via its nuclear basket interaction domain, which is made up of two zinc-finger containing modules ( Gunkel *et al.*, 2023). A yeast homolog of ZC3HC1, known as Pml39, has a nuclear basket interaction domain with a similar structure to the human protein, but a low degree of sequence similarity ( Gunkel *et al.*, 2023). Pml39 is involved in the retention of improper messenger ribonucleoparticles in the nucleus ( Palancade *et al.*, 2005), suggesting that ZC3HC1 could play a role similar to TPR in regulating mRNA export. The precise position of Pml39/ZC3HC1 in the nuclear basket has yet to be determined ( Singh *et al.*, 2024)

Here we show that, in primary human fibroblasts, although ZC3HC1 localises at the NPC and that this localisation depends on TPR, the localisation of TPR to nuclear pores is not ZC3HC1 dependent, contrary to previous reports in other cells. Consistent with this, we show that knockdown of ZC3HC1 in fibroblasts during OIS does not affect the heterochromatin re-organisation that manifests as the formation of SAHF, or the activation of the senescence secretory-associated phenotype (SASP), and that we have previously shown to be dependent on the localization of TPR at the nuclear basket ( Boumendil *et al*., 2019). Furthermore, knockdown of ZC3HC1 in fibroblasts produces a transcriptional signature that only partially overlaps that seen after TPR knockdown. Together, these data suggest that TPR and ZC3HC1 have different functions in human fibroblasts.

## Methods

### Cell culture and siRNA transfection

Human IMR90 cells, infected with pLNC-ER:STOP retroviral vectors to produce neomycin resistant control cells (IMR90-STOP) ( Acosta *et al.*, 2013), were cultured in DMEM with 10% FBS, 100 nM 4-hydroxytamoxifen (4-OHT) and 1% penicillin/streptomycin in a 37 °C incubator with 5% CO _2_.

IMR90 cells were similarly infected with pLNC-ER:RAS retroviral vectors to generate IMR90-RAS cells. Incubation in media containing 100 nM 4-hydroxytamoxifen was used to induce OIS in these cells as previously described ( Acosta *et al*., 2013; Bartlett *et al*., 2024; Boumendil *et al*., 2019).

siRNA knockdown was carried out as previously described ( Bartlett *et al.*, 2024; Boumendil *et al.*, 2019). Briefly, 9 × 10 ^5^ STOP IMR90 cells (except for imaging experiments, which used 1.5 × 10 ^5^ cells) were transfected using Dharmafect transfection reagent (Dharmacon) with a 30nM final concentration of control (siCTRL, D-001810-10-59) or TPR (siTPR, L-010548-00) or ZC3HC1 (siZC3HC1, L-016879-02) siRNA pools (Dharmacon) ( Table 1). Transfections were carried out in the presence of 4-OHT and were repeated at day 0, 3 and 6 and cells fixed for imaging on day 8 ( Figure 1B). For RNA-seq, cells were harvested after 3 days after siRNA transfection.

OIS was induced in IMR90-RAS cells using 100nM 4-OHT for 8 days, and treated with siRNAS (control, TPR and ZC3HC1), as previously described ( Boumendil *et al*., 2019).

### Immunofluorescence

Cells grown on coverslips were fixed with 4% paraformaldehyde and blocked with 1% bovine serum albumin (BSA) as previously described ( Bartlett *et al.*, 2024). Coverslips were then incubated with primary antibody diluted in 1% BSA at the dilutions detailed in Table 1, for 45 mins in a humid chamber. After washing three times with PBS, coverslips were then incubated for 30 mins with fluorescently labelled secondary antibodies (Life Technologies, Table 1) followed by two washes in PBS. PBS with 50ng/ml DAPI was added for 4 mins, before a final wash with PBS and mounting onto slides with VectaShield (Vector Laboratories).

**Table 1.** List of antibodies and siRNAs. Antibodies used in immunofluorescence (IF) experiments with their corresponding dilutions. RRIDs are from https://www.rrids.org. Catalog numbers for SiRNA pools (Dharmacon) are also listed.

	Source or reference	Identifiers	Dilution
anti-TPR (rabbit polyclonal)	Abcam. Raised against residues 2300-2349	ab84516 RRID:AB_1861454	IF (1:500)
anti-TPR (mouse monoclonal)	Gift from Volker Cordes. Raised against residues 1462- 1500		IF (1:100)
anti-ZC3HC1 (mouse monoclonal)	Santa Cruz. Raised against residues 1-300	sc-365058 RRID:AB_10847677	IF (1:100)
anti-ZC3HC1 (guinea pig polyclonal)	Gift from Volker Cordes. Raised against residues 307-355		IF (1:200)
Anti-IL1b ((mouse monoclonal)	Bio-techne. Raised against Recombinant human IL-1 beta/IL-1F2 aa residues 117-269	MAB 201 RRID:AB_358006	IF (1:100)
anti-mouse IgG (H+L) secondary, Alexa Fluor 568 (donkey polyclonal)	Invitrogen	A10037 RRID:AB_11180865	IF (1:1000)
anti-rabbit IgG (H+L) secondary, Alexa Fluor 488 (goat polyclonal)	Invitrogen	A11034 RRID:AB_2576217	IF (1:1000)
On target plus non targeting control	Dharmacon	D-001810-10-59	
ON-TARGETplus Human siZC3HC1 pool	Dharmacon	L-016879-02	
ON-TARGETplus Human siTPR pool GAAGAAGUGCGUAAGAAUA UCAGUUGACUCCAGGAAUA UCAAGGAGGUUUAGGAAUG GGCAUACACUUACUAGAAA	Dharmacon	L-010548-00	

Epifluorescence images were acquired as previously described ( Bartlett *et al.*, 2024). Super-resolution images were acquired by Instant Sim microscopy ( Azuma & Kei, 2015) using a Nikon SoRa™ system. Imaging was carried out using a SR HP Plan Apo λS 100x 1.35NA Silicone lens (Nikon Instruments). The CMOS cameras used for acquisition were Teledyne Photometrics Prime 95B 488 / 561nm laser lines. Step size for Z stacks was set to 0.120μm as required by manufacturer’s software. Acquisition of images and deconvolution was carried out using the Nikon NIS Elements Advanced Research software ( https://www.microscope.healthcare.nikon.com/products/software/nis-elements/software-resources).

***Quantitative reverse transcription polymerase chain reaction (RT-qPCR).*** Total RNA was extracted using the RNeasy mini kit (Qiagen) and cDNAs generated using SuperScript II (Life Technologies). Real-time PCR was performed on a Bio-Rad CFX Touch using SYBR Green PCR master mix (Roche) and the primers specific for *GAPDH* (fw: CAGCCTCAAGATCATCAGCA; rev: TGTGGTCATGAGTCCTTCCA) and IL-1 *β* (fw:TGCACGCTCCGGGACTCACA; rev: CATGGAGAACACCACTTGTTGCTCC). A series of cDNA solutions with known dilutions were incubated with each set of primers to obtain relative expression values for each sample. Expression was normalised to *GAPDH*.

### RNA-seq library preparation and analysis

Total RNA was extracted from a 10cm tissue culture plate using the RNeasy mini kit (Qiagen). Library preparation, sequencing and data quality control were carried out as previously described ( Bartlett *et al.*, 2024).

Differential expression analysis was carried out using DeSeq2 ( Love *et al.*, 2014). Gene ontology analysis was carried out using clusterProfiler ( Wu *et al.*, 2021). Volcano plots were rendered using ggplot2 ( Wickham, 2016) and Venn diagrams rendered using VennDiagram ( Chen & Boutros, 2011). A list of intronless genes was obtained from the UCSC hg19 GTF file ( Nassar *et al.*, 2023) by sorting for genes with a single exon. The list of histone genes was obtained from HGNC ( Braschi *et al.*, 2019).

***Image analysis.*** For the quantification of SAHF, images were put through a macro in ImageJ in order to blind sample file names. Blinded images were assessed in the DAPI channel for the presence of SAHF, before plotting unblinded data using Graphpad Prism v9.0.

### Statistics

Statistical analysis for Figure 3A and Figure 4 were performed using R and the specific statistical tests used are described in the relevant text and Figure legends. For Figure 3D, a one-sided Fisher’s exact test was used to assess whether the overlap between genes dysregulated upon TPR or ZC3HC1 knockdown exceeds chance.

### Data availability

RNA-seq data for TPR and ZC3HC1 are available from NCBI GEO under Accession numbers GSE264387 and GSE286436 respectively.

## Results

### ZC3HC1 localisation to nuclear pores is dependent on TPR but ZC3HC1 knockdown does not delocalise TPR

We first sought to verify the presence of ZC3HC1 at nuclear pores. We used siRNAs to deplete TPR or ZC3HC1 in human IMR90 fibroblasts ( Bartlett *et al.*, 2024) over an 8-day period ( Figure 1B). Immunofluorescence and wide-field epifluorescence microscopy showed both TPR and ZC3HC1 present at the nuclear periphery in cells transfected with control siRNA ( Figure 1C). Depleting TPR caused ZC3HC1 to move away from the nuclear periphery and into the nucleoplasm as previously reported ( Gunkel *et al.*, 2021). However, TPR appeared to remain concentrated at the nuclear periphery in cells visibly depleted of ZC3HC1, though we cannot exclude that there may still be small residual amounts of ZC3HC1 remaining after siRNA-mediated knockdown. Super-resolution microscopy showed TPR and ZC3HC1 colocalised at nuclear pores in cells treated with the control siRNA ( Figure 1D). Although TPR knockdown caused ZC3HC1 to move away from the nuclear periphery, TPR remained visibly localised to nuclear pores when ZC3HC1 was knocked down ( Figure 1D).

### ZC3HC1 knockdown does not affect the formation of SAHF or the senescence-associated secretory phenotype during OIS

**Figure 2.**
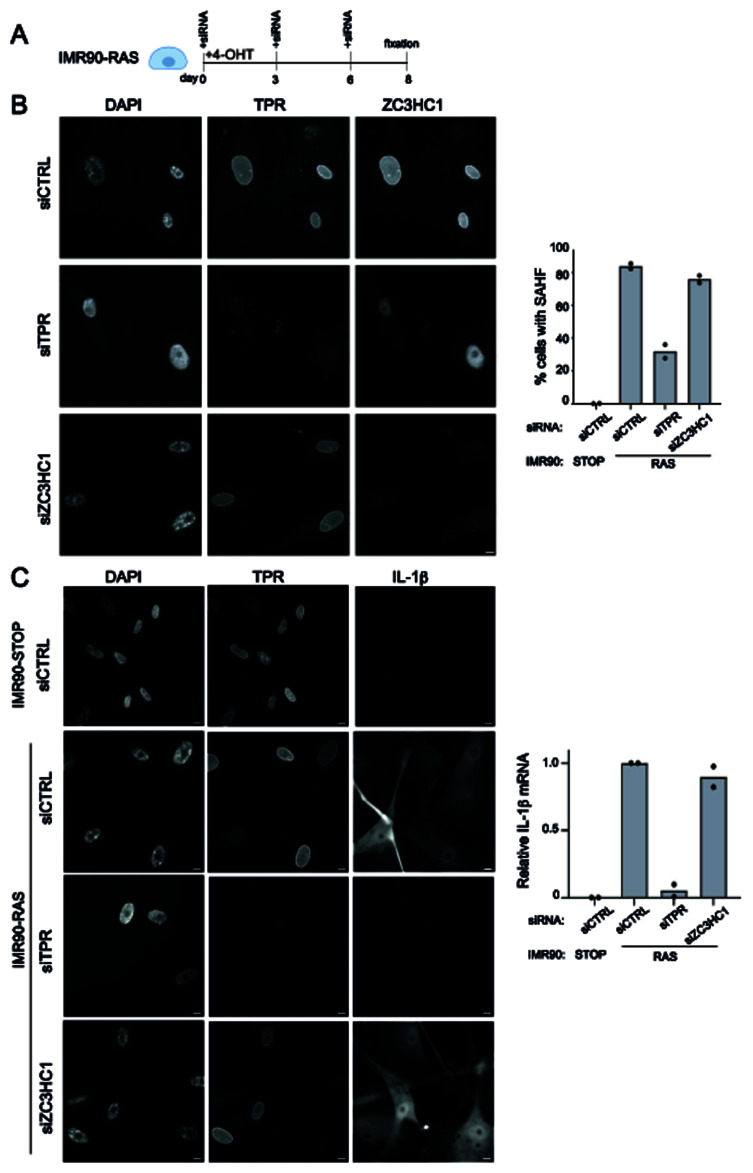
ZC3HC1 knockdown does not affect SAHF or the SASP during senescence. **A**) Schematic of experimental protocol for ZC3HC1 knockdown in IMR90-RAS cells induced into senescence with 4OHT. **B**) Left: TPR and ZC3HC1 immunostaining in senescent IMR90-RAS DAPI-stained cells after 4-OHT and siRNA treatment for 8 days, with antibodies detecting TPR (Abcam ab84516) or ZC3HC1 (gift from Volker Cordes and Philip Gunkel). SAHF are detected as bright DAPI-positive foci in the nucleoplasm. Scale bar = 10μm. Right: Mean percentage of cells containing SAHF in two biological replicates of IMR90-STOP and IMR90-RAS cells treated for 8 days with 4-OHT and control (siCTRL), TPR or ZC3HC1 siRNAs. **C**) Left: immunostaining for TPR and IL-1β in DAPI-stained IMR90-STOP cells and in IMR90-RAS cells after 4-OHT and treatment with control (CTRL) siRNA or with siRNAs targeting TPR or ZC3HC1 for 8 days. Scale bar = 10μm. Right: RT-qPCR for *IL-1β* in STOP cells and RAS cells treated with control (CTRL) siRNA or with siRNAs targeting TPR or ZC3HC1. Expression is relative to RAS cells treated with siCTRL and normalised to *GAPDH* expression.

We have previously shown that the presence of TPR at the nuclear basket is required for heterochromatin reorganisation (SAHF formation), and the inflammatory signalling known as the senescence-associated secretory phenotype (SASP), during oncogene-induced senescence in IMR90 cells induced to express an oncogenic form of RAS (RAS ^G12V^) ( Bartlett *et al*., 2024; Boumendil *et al*., 2019). If ZC3HC1 depletion leads to loss of TPR at the nuclear basket, we might therefore expect to see an impact on SAHF formation and the SASP during OIS. To investigate this, we treated RAS cells with 4-OHT for 8 days and transfected with either a control siRNA (siCTRL) or siRNAs depleting TPR or ZC3HC1 ( Figure 2A). SAHF were readily detected in control siRNA transfected IMR90-RAS cells and, consistent with our previous data ( Bartlett *et al*., 2024; Boumendil *et al*., 2019), SAHF did not form in RAS cells transfected with the TPR siRNAs ( Figure 2B). However, SAHF were present in cells depleted of ZC3HC1 (siZC3HC1) with no significant difference in the percentage of cells with SAHF between the RAS siCTRL and RAS siZC3HC1 samples ( Figure 2B). Immunostaining for IL-1β, a SASP cytokine showed, as expected, loss of IL-1β production in IMR90-RAS cells after depletion of TPR, but IL-1β was readily detected in the cytoplasm of IMR90-RAS cells transfected with siRNAs targeting ZC3HC1 ( Figure 2C). This suggests that the SASP is not affected by ZC3HC1 knockdown. These results were verified by RT-qPCR for *IL-1β* mRNA. Knocking down TPR led to a significant decrease in *IL-1β* expression but there was no difference in *IL-1β* mRNA levels between senescent cells treated with the control or ZC3HC1 siRNAs ( Figure 2C).

### The transcriptional signatures of ZC3HC1 and TPR knockdown are distinct

To better differentiate the functions of ZC3HC1 and TPR, we examined whether the transcriptional changes upon ZC3HC1 knockdown are similar to those that we have reported upon knockdown of TPR in IMR90 cells ( Bartlett *et al.*, 2024). We carried out RNA-seq on IMR90 fibroblasts treated with either control (siCTRL) or ZC3HC1 siRNAs for three days, using the same protocol as for TPR knockdown ( Bartlett *et al.*, 2024).

**Figure 3.**
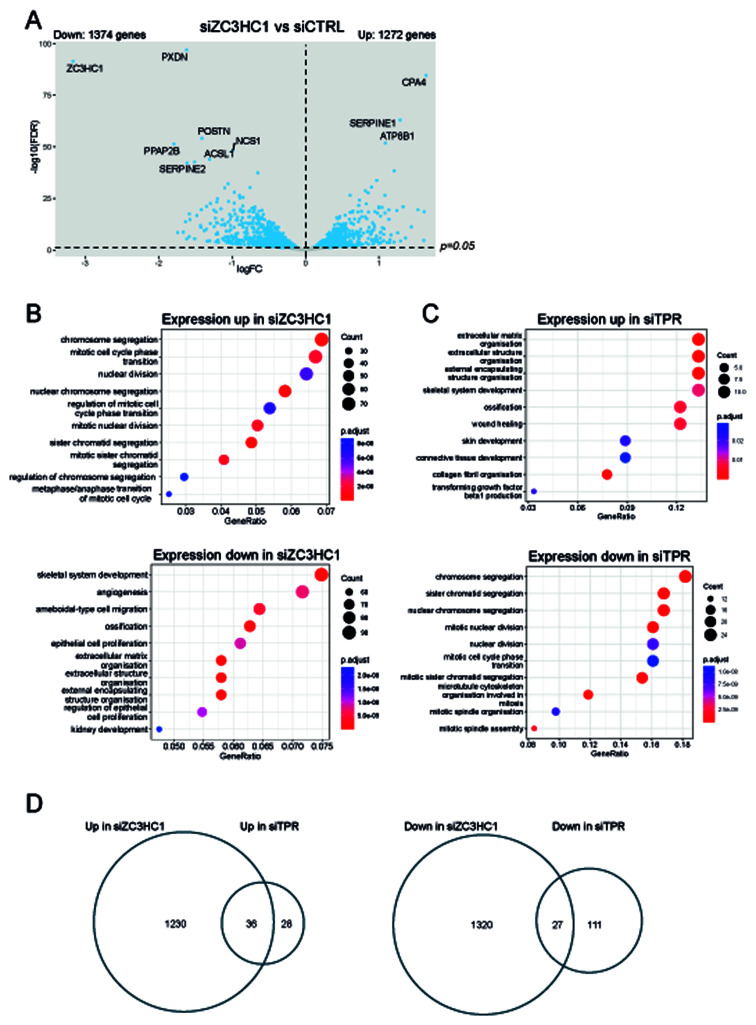
TPR and ZC3HC1 knockdown have distinct transcriptional signatures. **A**) Volcano plots showing differential expression analysis comparing siZC3HC1 and siCTRL samples. IMR90 cells were treated with the indicated siRNAs for 3 days. Blue dots indicate differentially expressed genes (adjusted p-value (FDR) < 0.05). The dashed horizontal line indicates an adjusted p-value of 0.05 and the dashed vertical line indicates a logFC of 0. The 10 genes with the most significant p-values are labelled. **B** and **C**) GO analysis carried out using clusterProfiler ( Wu *et al.*, 2021) for genes which increase or decrease in expression when ( **B**) ZC3HC1 or ( **C**) TPR is knocked down. RNA-seq data for TPR knockdown is taken from ( Bartlett *et al.*, 2024). **D**) Venn diagrams representing the overlaps between significantly up or downregulated transcripts upon TPR or ZC3HC1 loss in IMR90 cells.

ZC3HC1 knockdown led to more extensive changes in gene expression than knocking down TPR ( Bartlett *et al.*, 2024), with 1272 genes upregulated and 1374 downregulated upon ZC3HC1 knockdown ( Figure 3A). The most downregulated gene was *ZC3HC1*, confirming successful knockdown. Gene ontology analysis showed significant differences between genes whose expression changes in response to either ZC3HC1 or TPR knockdown ( Figure 3B and C). Loss of ZC3HC1 led to upregulation of genes associated with chromosome segregation and mitosis, consistent with the known role of ZC3HC1 in degrading cyclin B1 ( Bassermann *et al.*, 2005). Genes associated with angiogenesis, skeletal system development and extracellular matrix organisation were downregulated upon ZC3HC1 knockdown ( Figure 3B). TPR knockdown showed the opposite: mRNAs for genes involved in chromosome segregation were downregulated and those associated with extracellular matrix organisation were upregulated ( Figure 3C). This may be consistent with the chromosome segregation defects reported in human cancer cell lines upon TPR knockdown ( Nakano *et al*., 2010).

To investigate whether the two nuclear basket proteins have any shared functions, we examined how many genes change in expression upon both TPR knockdown and ZC3HC1 knockdown. Thirty-six (2.8%) of the 1236 genes significantly upregulated upon ZC3HC1 knockdown were also upregulated on TPR knockdown (64 genes upregulated in siTPR in total). Despite the relatively small number (36) of shared upregulated genes, this is a higher degree of overlap than expected by change (Odds ratio 60.69; p=2.74e-43). Twenty seven (2.0%) of the 1347 genes downregulated upon ZC3HC1 knockdown were also downregulated on TPR knockdown (138 genes downregulated in total upon TPR knockdown). This is also a higher degree of overlap than expected by chance (Odds ratio 10.40; p=1.88e-17) ( Figure 3D). The limited, but significant, especially for upregulated genes, suggests that TPR and ZC3HC1 have some overlapping, but also many distinct, functions in terms of gene expression and mRNA metabolism.

### ZC3HC1 is not required for the export of mRNAs from intronless genes

**Figure 4.**
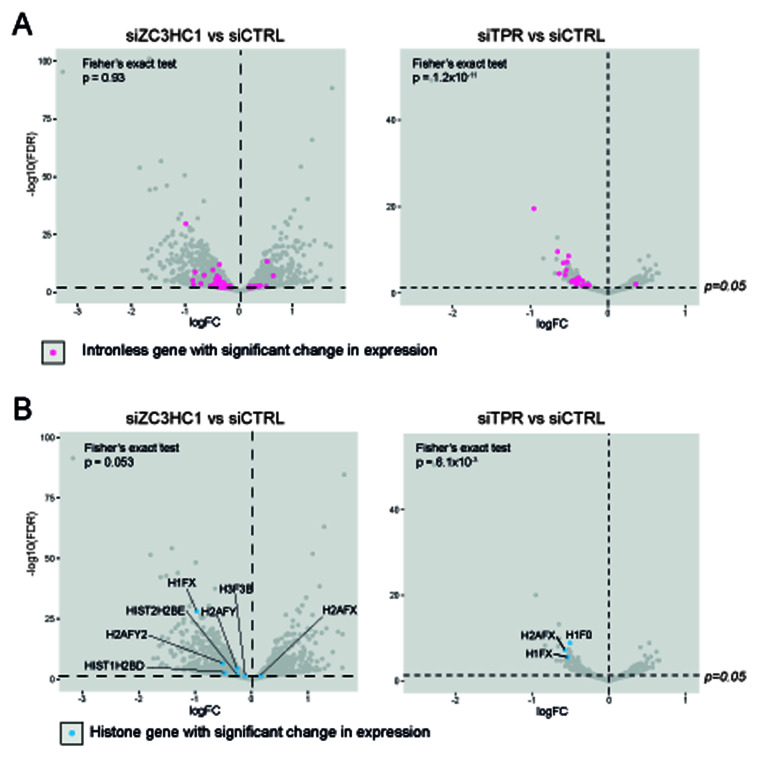
ZC3HC1 knockdown does not affect the expression of intronless or histone genes. **A**) Volcano plots of differential expression analysis of IMR90 cells treated with CTRL vs ZC3HC1 siRNAs (left), or CTRL vs TPR siRNAs (right). Intronless genes are labelled in pink. The horizontal dashed line indicates an adjusted p-value (FDR) of 0.05 and the vertical dashed line indicates a logFC of 0. Fisher’s exact tests were carried out to determine whether the number of downregulated intronless genes was greater than expected by chance. **B**) As in ( **A**) but with histone genes labelled in blue. Fisher’s exact tests were carried out to determine whether the number of downregulated histone genes was greater than expected by chance.

Consistent with the known role of TPR in the nuclear export of short and intronless mRNAs via interaction with the TREX-2 complex, TPR knockdown causes significant downregulation of mRNAs from intronless and histone genes ( Aksenova *et al.*, 2020; Bartlett *et al.*, 2024; Lee *et al.*, 2020; Zuckerman *et al.*, 2020). We investigated whether ZC3HC1, by localizing TPR at the nuclear basket, might play a similar role in mRNA nuclear export. Fisher’s exact tests showed that, in contrast to TPR knockdown, there were no more mRNAs for intronless genes significantly downregulated upon ZC3HC1 knockdown than would be expected by chance (p=0.93) ( Figure 4A). There were also no more histone mRNAs downregulated upon ZC3HC1 knockdown than would be expected by chance (p=0.053) ( Figure 4B). This suggests that, unlike TPR, ZC3HC1 does not have a role in the nuclear export of intronless mRNAs.

## Discussion

We have confirmed that ZC3HC1 localises to the nuclear periphery, suggesting that it is a nuclear pore protein, as reported ( Gunkel *et al.*, 2021). However, depleting ZC3HC1 in IMR90 cells did not appear to affect the localisation of TPR to nuclear pores, as assessed by immunofluorescence, contrary to previous reports ( Gunkel *et al.*, 2021; Gunkel & Cordes, 2022) ( Figure 1). This suggests that, at least in human fibroblasts, ZC3HC1 may not play the structural role in establishing interconnections between TPR polypeptides at the nuclear basket which has been reported in cancer cells ( Gunkel & Cordes, 2022). In support of this, we show that depletion of ZC3HC1 during oncogene-induced senescence in IMR90-RAS cells does not affect formation of SAHF or induction of the SASP, two OIS cell phenotypes that we have previously shown are dependent on TPR being located at the nuclear pore basket ( Bartlett *et al*., 2024; Boumendil *et al*., 2019) ( Figure 2). NPC composition is known to vary between cell types, levels of *TPR* expression also vary between human tissues, and mutation of nuclear pore basket proteins can result in tissue-specific phenotypes ( Guglielmi *et al*., 2020; Ori *et al*., 2013). In the future it will be interesting to investigate whether these contrasting results about the dependence of TPR localization on ZC3HC1 reflect differences in nuclear pore baskets between cell types.

We did confirm that TPR is required for the localisation of ZC3HC1 to nuclear pores in IMR90 fibroblasts ( Figure 1). Even though ZC3HC1 is displaced to the nucleoplasm on TPR knockdown, only a small number of mRNAs which we previously reported changed in expression after TPR knockdown ( Bartlett *et al.*, 2024) also change in expression upon ZC3HC1 knockdown ( Figure 3). This suggests that most mRNAs that change in expression in response to reduction of ZC3HC1 do not depend on its localisation at the nuclear basket. Amongst the mRNAs downregulated upon ZC3HC1 depletion, we do not see an enrichment of mRNAs originating from intronless or histone genes, which are known to dependent on TPR localisation at the nuclear pore basket for their nuclear export ( Aksenova *et al.*, 2020; Bartlett *et al.*, 2024; Lee *et al.*, 2020) ( Figure 4). This further supports our conclusion that ZC3HC1 is not required for TPR localisation to the nuclear pore basket in IMR90 cells.

Our RNA-seq data showing that levels of mRNAs involved in chromosome segregation and the mitotic cell-cycle phase are elevated on ZC3HC1 knockdown is consistent with a role for ZC3HC1 in the regulation of mitosis. There is a report that ZC3HC1 is a component of a nuclear SCF E3 ligase, which is required for the degradation of cyclin B1 and thereby regulates mitotic entry and exit ( Bassermann *et al.*, 2005), however other reports do not support a role for ZC3HC1 as a SCF E3 ligase ( Gunkel *et al*., 2021; Gunkel *et al*., 2023). To distinguish which functions of ZC3HC1 are dependent on its localisation to the nuclear pore, RNA-seq could be repeated in cells expressing a version of ZC3HC1 which cannot localise to nuclear pores, for example by making a single amino acid substitution in ZC3HC1 (C429S) which abolishes its interaction with TPR ( Gunkel & Cordes, 2022).

## Ethics and consent

Ethical approval and consent were not required

## Data Availability

All quantitative data associated with this manuscript are been deposited in NCBI GEO and is freely accessible with no restrictions on use or distribution. NCBI GEO: RNA-seq data following TPR knockdown in IMR90 cells. GSE264387; https://www.ncbi.nlm.nih.gov/geo/query/acc.cgi?acc=GSE264387 ( Bartlett *et al.*, 2024) NCBI GEO: RNA-seq data following ZC3H1 knockdown in IMR90 cells. GSE286436; https://www.ncbi.nlm.nih.gov/geo/query/acc.cgi?acc=GSE286436 ( Bartlett & Bickmore, 2025)
