# Supplementary material for: ZC3HC1 has many functions distinct from TPR and is dispensable for TPR localisation to the nuclear basket
Source: Wellcome Open Res. 2025 Nov 24;10:188. Originally published 2025 Apr 11. [Version 2] doi: 10.12688/wellcomeopenres.23711.2 (PMC12120416; doi:10.12688/wellcomeopenres.23711.2)
Supplement: Peer review history

# Reviewer report: Reviewer response for version 2

**DOI:** 10.21956/wellcomeopenres.27985.r140542

**Published:** 2025-12-29

**Authors:** Alexander Palazzo

**Affiliations:** 1 Department of Biochemistry, University of Toronto, Toronto, ON, M5G 1M1, Canada

The authors address several concerns, although I still believe they over-interpret their data. The observation that many TPR-dependent mRNAs are also ZC3HC1-dependent suggests that these factors may act within the same pathway. However, the fact that many ZC3HC1-dependent mRNAs are not among the TPR-dependent set is unsurprising, given that relatively few mRNAs exhibit clear TPR dependency. It is possible that the overlap would be greater if the authors examined mRNA export rather than total mRNA levels.

 The authors have also included new data addressing the requirement for ZC3HC1 in TPR-dependent processes such as heterochromatin reorganization (SAHF formation) and inflammatory signaling. Because the extent of ZC3HC1 depletion remains unclear, the authors should again temper their conclusions—could residual ZC3HC1 protein be sufficient to maintain these processes?

Is the work clearly and accurately presented and does it cite the current literature?

Yes

If applicable, is the statistical analysis and its interpretation appropriate?

Partly

Are all the source data underlying the results available to ensure full reproducibility?

Yes

Is the study design appropriate and is the work technically sound?

Yes

Are the conclusions drawn adequately supported by the results?

Partly

Are sufficient details of methods and analysis provided to allow replication by others?

Yes

Reviewer Expertise:

mRNA nuclear export in mammalian cells.

I confirm that I have read this submission and believe that I have an appropriate level of expertise to confirm that it is of an acceptable scientific standard, however I have significant reservations, as outlined above.

# Reviewer report: Reviewer response for version 2

**DOI:** 10.21956/wellcomeopenres.27985.r140538

**Published:** 2025-12-11

**Authors:** Richard W. Wong

**Affiliations:** 1 Kanazawa University, Kanazawa, Japan

I’ve reviewed the revised version and the additional data and analyses. I agree with the revisions and am happy to update my status to Approved.

Is the work clearly and accurately presented and does it cite the current literature?

No

If applicable, is the statistical analysis and its interpretation appropriate?

Partly

Are all the source data underlying the results available to ensure full reproducibility?

Yes

Is the study design appropriate and is the work technically sound?

Yes

Are the conclusions drawn adequately supported by the results?

No

Are sufficient details of methods and analysis provided to allow replication by others?

Yes

Reviewer Expertise:

NA

I confirm that I have read this submission and believe that I have an appropriate level of expertise to confirm that it is of an acceptable scientific standard.

# Reviewer report: Reviewer response for version 2

**DOI:** 10.21956/wellcomeopenres.27985.r140540

**Published:** 2025-12-11

**Authors:** Michael Rout

**Affiliations:** 1 Rockefeller University, New York, USA

The manuscript is acceptable in its current form.

Is the work clearly and accurately presented and does it cite the current literature?

Yes

If applicable, is the statistical analysis and its interpretation appropriate?

Yes

Are all the source data underlying the results available to ensure full reproducibility?

Yes

Is the study design appropriate and is the work technically sound?

Yes

Are the conclusions drawn adequately supported by the results?

Yes

Are sufficient details of methods and analysis provided to allow replication by others?

Yes

Reviewer Expertise:

NA

I confirm that I have read this submission and believe that I have an appropriate level of expertise to confirm that it is of an acceptable scientific standard.

# Reviewer report: Reviewer response for version 1

**DOI:** 10.21956/wellcomeopenres.26159.r122276

**Published:** 2025-06-12

**Authors:** Michael Rout, Chris Caffalette, Trevor van van Eeeuwen

**Affiliations:** 1 Rockefeller University, New York, USA

Bickmore et al have looked at the role of ZC3HC1 in the organization of the nuclear basket feature of the human nuclear pore complex (NPC). The nuclear basket is a structural and functional nucleoplasmic extension of the NPC, fundamentally comprised of TPR, Nup153, and Nup50 in metazoans. Bickmore et al investigated the structural dependence of the nuclear basket filament protein TPR on the presence of ZC3HC1, finding limited evidence for the failure of TPR to localize to the nuclear periphery upon ZC3HC1 siRNA knockdown. This finding is in stark contrast to the body of work from Gunkel et al. (cited in the manuscript) that has shown a subpopulation of TPR depends on ZC3HC1 for localization to the nuclear periphery. An important feature of this preprint is the use of primary human fibroblasts, rather than cancer cell lines, to demonstrate this finding, which may yield a new result because it reflects normal homeostatic behavior of ZC3HC1 in a non-cancer human cell line. Further, Bickmore et al performed RNA-seq on IMR90 fibroblasts following ZC3HC1 and TPR siRNA knockdowns to investigate changes in gene expression. Analysis of genes upregulated or downregulated showed that TPR and ZC3HC1 have distinct functions in the cell, highlighting that in contrast to TPR, ZC3HC1 is not crucial to the export of intronless mRNAs from the nucleus. 

 The following is a list of Major Points and Minor Points for the authors to consider: 

**Major Points: **

1. Figure 2 shows that ZC3HC1 transcripts are the most downregulated species following siRNA knockdown, leading to the statement “ *depleting ZC3HC1 in IMR90 cells did not affect the localisation of TPR to nuclear pores, as assessed by immunofluorescence, contrary to previous reports (Gunkel et al., 2021; Gunkel & Cordes, 2022).*”  By contrast, in Figure 1D, immunofluorescence imaging of ZC3HC1 in the siZC3HC1 panel shows signal for ZC3HC1 both in the nuclear interior as well as a nuclear rim-staining pattern. The authors may consider a Western blot to validate changes in protein level (to complement RNAseq analysis) due to considerations of protein half-life and turnover. 

1. While the differences from Gunkel et al 2021, 2023 have been discussed in the context of cell line differences, it could also benefit the reader to include additional exploration of possible mechanisms behind the alternative results. Additionally, the authors may also consider including additional images, similar to Figure 1D, in a supplement. These additions may help the reader understand the support for the conclusions drawn regarding the behavior of TPR following ZC3CH1 knockdown in the IMR90 cells and their potential biological underpinnings. 

1. It may be beneficial to the discussion of the results to consider the work of Gunkel et al that did not find evidence for ZC3HC1 as a nuclear SCF E3 ligase (Gunkel et al 2021, Gunkel et al 2023) as this would be both interesting and does not diminish the conclusions from the RNA-seq data. 

 

**Minor Points:**

 None noted.

Is the work clearly and accurately presented and does it cite the current literature?

Yes

If applicable, is the statistical analysis and its interpretation appropriate?

Yes

Are all the source data underlying the results available to ensure full reproducibility?

Yes

Is the study design appropriate and is the work technically sound?

Yes

Are the conclusions drawn adequately supported by the results?

Yes

Are sufficient details of methods and analysis provided to allow replication by others?

Yes

Reviewer Expertise:

Nuclear cell biology, nuclear pore complexes, nuclear envelope, nuclear transport.

We confirm that we have read this submission and believe that we have an appropriate level of expertise to confirm that it is of an acceptable scientific standard, however we have significant reservations, as outlined above.

# Reviewer report: Reviewer response for version 1

**DOI:** 10.21956/wellcomeopenres.26159.r122284

**Published:** 2025-05-29

**Authors:** Richard W. Wong

**Affiliations:** 1 Kanazawa University, Kanazawa, Japan

Reviewer Comments (Major Revision Required): 

 The manuscript by Bartlett et al. presents a careful comparative study on the nuclear basket proteins ZC3HC1 and TPR, showing that ZC3HC1 requires TPR for nuclear pore localisation, but not vice versa, and that their knockdowns result in distinct transcriptional profiles. The work is well conducted and addresses an important question in nuclear pore complex (NPC) biology. However, I have several concerns that must be addressed before this manuscript can be considered for acceptance. 

 1. Depth of Discussion on TPR Multifunctionality:****

 While the manuscript focuses primarily on TPR’s role in mRNA export and heterochromatin exclusion, TPR’s broader functions, especially its connections to mitotic regulation, autophagy, and tumorigenesis, are not sufficiently acknowledged. Several previous studies have demonstrated that TPR interacts with mitotic regulators (e.g., Aurora A kinase), is involved in sustaining mitosis via dynein interaction, and plays critical roles in autophagy regulation and cancer cell survival. These aspects are highly relevant to the findings on ZC3HC1 and mitosis-related gene expression changes. Therefore, I request that the authors integrate and cite the following foundational studies on TPR functions: 

- Bartlett B, et al. (2024) - **Ref 1**
- Dewi F, et al. (2021) - **Ref 2**
- Singh D, Soni N, et al. (2024) - **Ref 3**
- Dewi F, Domoto T, et al. (2024) - **Ref 4**
- Gunkel P, et al. (2023) - **Ref 5**
- Rajanala K,et al. (2022) - **Ref 6**
- Kobayashi A, et al. (2015) - **Ref 7 **
- Li Y, et al. (2021) - **Ref 8**
- Funasaka T, et al. (2021) - **Ref 9**
- Kosar M, et al. (2021) - **Ref 10**
- Nakano H, et al. (2010) - **Ref 11**
- Lee E, et al. (2020) - **Ref 12**

 

 Discussing these studies would provide a broader biological context for the distinct mitotic and autophagy-related signatures observed upon ZC3HC1 depletion, especially with Volker Cordes’s group published ZC3HC1 findings. 

 2. Further Statistical Validation: 

 As another reviewer already noted, the observed overlap in gene sets between TPR and ZC3HC1 knockdowns should be statistically evaluated against random expectation. Please apply appropriate statistical tests (e.g., hypergeometric test) to rigorously assess whether the overlap is truly non-significant. 

 3. Clarification of ZC3HC1 and TPR Knockdown Efficiency: 

 While immunofluorescence data suggest efficient knockdown, an immunoblot for ZC3HC1 (and ideally TPR) should be provided. Even residual low levels could potentially sustain partial nuclear basket architecture, impacting interpretation. 

 4. Expansion of mRNA Characteristic Analyses: 

 I encourage the authors to perform additional analyses on the characteristics (e.g., GC content, length, intron content) of the mRNAs affected by ZC3HC1 depletion, as suggested by previous reviews. This could shed light on whether ZC3HC1 loss preferentially affects certain classes of transcripts. 

 5. siRNA Information: 

 Full siRNA sequences must be listed in the methods section to ensure reproducibility, considering commercial catalog numbers may become obsolete. 

 6. Toning Down Some Statements: 

 Certain conclusions should be made more precise. For example, modify statements such as “TPR and ZC3HC1 have distinct functions” to “distinct functions in terms of gene expression and mRNA metabolism,” unless additional evidence beyond transcriptomics is provided.

Is the work clearly and accurately presented and does it cite the current literature?

No

If applicable, is the statistical analysis and its interpretation appropriate?

Partly

Are all the source data underlying the results available to ensure full reproducibility?

Yes

Is the study design appropriate and is the work technically sound?

Yes

Are the conclusions drawn adequately supported by the results?

No

Are sufficient details of methods and analysis provided to allow replication by others?

Yes

Reviewer Expertise:

NA

I confirm that I have read this submission and believe that I have an appropriate level of expertise to confirm that it is of an acceptable scientific standard, however I have significant reservations, as outlined above.

## References

[1] : TPR is required for cytoplasmic chromatin fragment formation during senescence. *eLife* .2024;13: 10.7554/eLife.101702 10.7554/eLife.101702 PMC1166624439625470

[2] : Nucleoporin TPR (translocated promoter region, nuclear basket protein) upregulation alters MTOR-HSF1 trails and suppresses autophagy induction in ependymoma. *Autophagy* .2021;17(4) : 10.1080/15548627.2020.1741318 1001-1012 10.1080/15548627.2020.1741318 PMC807876232207633

[3] : The molecular architecture of the nuclear basket. *Cell* .2024;187(19) : 10.1016/j.cell.2024.07.020 5267-5281.e13 10.1016/j.cell.2024.07.020 PMC1141631639127037

[4] : Colorectal cancer cells require glycogen synthase kinase-3β for sustaining mitosis via translocated promoter region (TPR)-dynein interaction. *Oncotarget* .2018;9(17) : 10.18632/oncotarget.24344 13337-13352 10.18632/oncotarget.24344 PMC586258229568361

[5] : An evolutionarily conserved bimodular domain anchors ZC3HC1 and its yeast homologue Pml39p to the nuclear basket. *Molecular Biology of the Cell* .2023;34(5) : 10.1091/mbc.E22-09-0402 10.1091/mbc.E22-09-0402 PMC1016241836857168

[6] : Correction: Phosphorylation of nucleoporin Tpr governs its differential localization and is required for its mitotic function. *Journal of Cell Science* .2022;135(6) : 10.1242/jcs.260004 10.1242/jcs.260004 PMC1103487435344051

[7] : Therapeutic potential of mitotic interaction between the nucleoporin Tpr and aurora kinase A. *Cell Cycle* .2015;14(9) : 10.1080/15384101.2015.1021518 1447-1458 10.1080/15384101.2015.1021518 PMC461490325789545

[8] : Distinct roles of nuclear basket proteins in directing the passage of mRNA through the nuclear pore. *Proceedings of the National Academy of Sciences* .2021;118(37) : 10.1073/pnas.2015621118 10.1073/pnas.2015621118 PMC844942234504007

[9] : Regulation of autophagy by nucleoporin Tpr. *Scientific Reports* .2012;2(1) : 10.1038/srep00878 10.1038/srep00878 PMC350182323170199

[10] : The human nucleoporin Tpr protects cells from RNA-mediated replication stress. *Nature Communications* .2021;12(1) : 10.1038/s41467-021-24224-3 10.1038/s41467-021-24224-3 PMC822580334168151

[11] : Nucleoporin Translocated Promoter Region (Tpr) Associates with Dynein Complex, Preventing Chromosome Lagging Formation during Mitosis. *Journal of Biological Chemistry* .2010;285(14) : 10.1074/jbc.M110.105890 10841-10849 10.1074/jbc.M110.105890 PMC285629020133940

[12] : TPR is required for the efficient nuclear export of mRNAs and lncRNAs from short and intron-poor genes. *Nucleic Acids Research* .2020;48(20) : 10.1093/nar/gkaa919 11645-11663 10.1093/nar/gkaa919 PMC767245833091126

# Reviewer report: Reviewer response for version 1

**DOI:** 10.21956/wellcomeopenres.26159.r122278

**Published:** 2025-05-29

**Authors:** Weidong Yang

**Affiliations:** 1 Temple University, Philadelphia, USA

This manuscript focuses on the nuclear basket protein ZC3HC1. The authors demonstrate that ZC3HC1’s localization within the nuclear basket depends on the presence of another nuclear basket component, TPR, whereas TPR’s localization is independent of ZC3HC1. They further show that ZC3HC1 knockdown does not affect the expression of intronless or histone genes. Instead, they suggest that ZC3HC1 may play a role in regulating mitosis.

 The study is relatively simple and straightforward, employing well-established methodologies. The data appear solid, and the conclusions are adequately supported.

Is the work clearly and accurately presented and does it cite the current literature?

Yes

If applicable, is the statistical analysis and its interpretation appropriate?

Yes

Are all the source data underlying the results available to ensure full reproducibility?

Yes

Is the study design appropriate and is the work technically sound?

Yes

Are the conclusions drawn adequately supported by the results?

Yes

Are sufficient details of methods and analysis provided to allow replication by others?

Yes

Reviewer Expertise:

Cell Biology

I confirm that I have read this submission and believe that I have an appropriate level of expertise to confirm that it is of an acceptable scientific standard.

# Reviewer report: Reviewer response for version 1

**DOI:** 10.21956/wellcomeopenres.26159.r122277

**Published:** 2025-05-10

**Authors:** Daniel Zenklusen

**Affiliations:** 1 Université de Montréal, Montréal, Canada

ZC3HC1 and TPR have been described as structural components of the nuclear basket. However, whether ZC3HC1’s function in basket biology is primarily structural or whether it has additional, and distinct, functions then TPR, has not been fully investigated. In this manuscript, Bartlett and coworkers use microscopy and RNomic approaches to propose that ZC3HC1 depletions in primary fibroblast don’t affect the level of TPR localized to the nuclear envelope, and that it affects expression of a distinct set of RNA when compared to TPR, suggesting distinct functions for these two basket proteins.

 Overall, the study is well designed, and their conclusions mostly supported by their data. The observation that TPR and ZC3HC1 depletions affect expression of different types of mRNA is intriguing and, as stated the authors, future mechanistic studies will be important to better understand the function of these two proteins at and off the NPC. However, the microscopy data in Figure 1 needs quantifications to allow the conclusions drawn by the authors, and/or a more balanced discussion with respect to previous studies.

 The authors perform siRNA KDs for ZC3HC1 and localize TPR signal using immunofluorescence to conclude that in IMR90 cells, TPR localization is not altered after ZC3HC1 depletion. However, the authors do not provide quantification of their data. As mentioned by the authors, previous studies by the Cordes lab suggested that only a fraction of NPC bound TPR requires ZC3HC1 for NPC/nuclear periphery association. Images shown in Figure 1C seem to show different intensities for nucleoplasmic and nuclear periphery localized TPR, as well as various expression levels of ZC3HC1 in different cells, suggesting different KD efficiencies in individual cells. To better support their conclusion, the authors have either to provide quantification that convincingly shows that ZC3HC1 depletions does not alter NE associated TPR levels, or provide a more nuanced interpretation of their data. Moreover, in case these quantifications show that IMR90 cells indeed do not contain a ZC3HC1 dependent fraction of TPR at the nuclear periphery, the authors might want to shortly discuss whether this might suggest that there t exist different types of baskets in different cell types, and refer to the appropriate literature related to NPC/basket specialization.

Is the work clearly and accurately presented and does it cite the current literature?

Yes

If applicable, is the statistical analysis and its interpretation appropriate?

Partly

Are all the source data underlying the results available to ensure full reproducibility?

Yes

Is the study design appropriate and is the work technically sound?

Yes

Are the conclusions drawn adequately supported by the results?

Partly

Are sufficient details of methods and analysis provided to allow replication by others?

Yes

Reviewer Expertise:

NPC, nuclear transport, microscopy

I confirm that I have read this submission and believe that I have an appropriate level of expertise to confirm that it is of an acceptable scientific standard, however I have significant reservations, as outlined above.

# Reviewer report: Reviewer response for version 1

**DOI:** 10.21956/wellcomeopenres.26159.r122280

**Published:** 2025-04-26

**Authors:** Alexander Palazzo

**Affiliations:** 1 Department of Biochemistry, University of Toronto, Toronto, ON, M5G 1M1, Canada

In this manuscript, Bartlett and co-authors invesigate the relationship between ZC3HC1 and TPR. They demonstrate that while the former requires the later for localization to the nuclear pore, the reverse is not true. They then analyze the effects of depleting each component on mRNA levels. The authors claim that these do not overlap, although some statistical assessment is warranted.  I only have a few concerns:

1. The authors should provide an immunoblot of ZC3HC1 – perhaps a low level of protein is sufficient for TPR localization? Although the authors may feel that this is unlikely, this possibility should be stated.
2. At the end of the transcriptomics section the authors state “The limited overlap in differentially expressed genes between the two knockdown experiments suggests that TPR and ZC3HC1 have distinct functions.” This should be toned down. If the two proteins function in protein trafficking or NPC assembly, this may not be reflected in changes in the transcriptome. They should at least modify the sentence by adding “distinct functions in terms of gene expression and mRNA metabolism.”
3. Although the overlap in Figure 2D is low in absolute counts, about 1/3rd of the mRNAs upregulated by TPR-depletion are found in the ZC3CH1-dependent set. The authors should determine whether this is above what is expect from chance alone. The same for the downregulated mRNAs. This will depend on the overall number of mRNAs that were evaluated in both sets. 
4. Some analysis of the mRNAs affected by ZC3HC1-depletion is warranted. Basic characteristics, such as GC-content, intron density, and mRNA length (as compared to the unaffected mRNAs) should be provided.
5. siRNA sequences should be listed – companies may stop listing items on their website and these sequences are important for reproducibility.

Is the work clearly and accurately presented and does it cite the current literature?

Yes

If applicable, is the statistical analysis and its interpretation appropriate?

Partly

Are all the source data underlying the results available to ensure full reproducibility?

Yes

Is the study design appropriate and is the work technically sound?

Yes

Are the conclusions drawn adequately supported by the results?

Partly

Are sufficient details of methods and analysis provided to allow replication by others?

Yes

Reviewer Expertise:

mRNA nuclear export in mammalian cells.

I confirm that I have read this submission and believe that I have an appropriate level of expertise to confirm that it is of an acceptable scientific standard, however I have significant reservations, as outlined above.
